# Treatment with Nasal Neuro-EPO Improves the Neurological, Cognitive, and Histological State in a Gerbil Model of Focal Ischemia

**DOI:** 10.1100/tsw.2010.215

**Published:** 2010-11-16

**Authors:** Yamila Rodríguez Cruz, Támos Yuneidys Mengana, Adriana Muñoz Cernuda, Nelvis Subirós Martines, Alina González-Quevedo, Iliana Sosa Testé, Julio César García Rodríguez

**Affiliations:** ^1^ Histology Department Preclinical and Basic Science Institute, Havana, Cuba; ^2^ National Center for Laboratory Animal Breeding, Havana, Cuba; ^3^ Center of Research and Development of Medicaments (CIDEM), Havana, Cuba; ^4^ Institute of Neurology and Neurosurgery (INN), Havana, Cuba; ^5^ Neuroscience Group of the National Center for Laboratory Animal Breeding (CENPALAB), Havana, Cuba

**Keywords:** ischemia, EPO, Neuro-EPO, nasal route, stroke, neuroprotection, gerbils

## Abstract

Vascular illness of the brain constitutes the third cause of death and the first cause of disability in Cuba and many other countries. Presently, no medication has been registered as a neuroprotector. Neuroprotection with intranasal Neuro-EPO (EPO, erythropoietin) has emerged as a multifunctional therapy that plays a significant role in neural survival and functional recovery in an animal model of stroke. On the other hand, there is limited access to the brain through the blood brain barrier (BBB) for intravenously applied EPO, and the high EPO dosages needed to obtain a protective effect increase the danger of elevated hematocrit levels and practically exclude chronic or subchronic treatment with EPO. A promising approach has been recently developed with a nonerythropoietic variant of EPO, Neuro-EPO, with low sialic acid content, a very short plasma half-life, and without erythropoietic activity, probably similar to endogenous brain EPO. The objective of this work was to determine the neuroprotective effect of intranasal Neuro-EPO in comparison with the human recombinant EPO injected intraperitoneally in the acute phase of cerebral ischemia, employing the common carotid artery occlusion model in gerbils. Neuro-EPO has demonstrated a better neuroprotective effect, evidenced through increased viability, improvements of the neurological state and cognitive functions, as well as protection of the CA3 region of the hippocampus, temporal cortex, and the thalamus. In conclusion, the intranasal application of Neuro-EPO has a better neuroprotective effect than intraperitoneal EPO, evidenced by the significant improvement of neurological, cognitive, and histological status in the animal model of stroke employed.

### INTRODUCTION

Cerebrovascular diseases (CVD) constitute the third cause of death and the first cause of disability for adults in Cuba[1] and in many developed countries[2]. The variant of greatest incidence is ischemic CVD, with a smaller mortality than hemorrhagic CVD, but with more impact on the loss of quality of life[2].

There are two specific therapeutic strategies for the treatment of acute ischemic stroke. The first is based on restoring the blood flow and the second is based on increasing the survival of the cells of the nerve tissue (neuroprotection) after the injury to the central nervous system (CNS).

Until now, there are no neuroprotectant drugs with sufficient efficacy and specificity for the treatment of acute stroke[3]. Many compounds with therapeutically promising results in animal studies have not had the same success in the clinical setting[4,5].

The use of endogenous biomolecules with therapeutic activity is a recent proposal in neuroscience research[6,7]. An example of this type of molecule is erythropoietin (EPO), a glycoprotein produced in the kidney and involved in the proliferation, differentiation, and maturation of erythrocyte progenitors, increasing oxygen supply to the tissues[8].

The observation that EPO and its receptor (EPO-R) are expressed in the brain has stimulated the development of studies related to the neuroprotective effect of this molecule in different models of stroke[9,10,11,12,13]. EPO expression increases during cerebral ischemia, suggesting its role in the endogenous neuroprotector system of the mammal's brain[14].

In the preclinical and clinical studies where recombinant human EPO (rHu-EPO) has been used as a neuroprotector, it has been delivered by different routes: intracerebroventricular (***ICV***), intraperitoneal (***IP***), and intravenous (***IV***)[15].

Employing the last two routes, this molecule reaches the bloodstream immediately, constituting a potential risk for the stimulation of hematopoiesis, with the subsequent increase of blood viscosity[16,17]. Therefore, the search for derivatives of EPO without significant hematopoietic activity, but conserving their neuroprotective properties, is a challenge for the investigators in this field[18]. The effectiveness of Neuro-EPO has been demonstrated in different models of damage to the nervous system[16].

One of the biggest difficulties for delivering therapeutic proteins to the nervous system is the blood brain barrier (BBB). However, the intranasal (***IN***) administration of therapeutic agents to the CNS has been proposed as a new alternative for delivering these to the CNS in diverse illnesses in humans[19] and animals[16]. ***IN*** drug administration could offer a noninvasive method that circumvents the BBB and provides rapid drug absorption for acute brain trauma without systemic side effects[18,20,21,22].

The nasal administration of rHu-EPO proved to be 10 times quicker than the ***IV*** route used for reaching the damaged brain[23]. Therefore, to stimulate endogenous neuroprotection occurring in the brain during a hypoxic insult, one could use an endogenous molecule similar to EPO (Neuro-EPO), administering it intranasally, as it is rapidly degraded in the liver.

In previous studies conducted in models of ischemia reperfusion in gerbils treated with ***IN*** Neuro-EPO[24] and using ***IN*** rHu-EPO in focal ischemia in rats[21,25], the effectiveness of their ***IN*** application has been demonstrated. The objective of this work was to determine the neuroprotector effect of the ***IN*** application of low sialic acid containing human recombinant erythropoietin (Neuro-EPO), and to compare it with rHu-EPO injected intraperitoneally ***(IP)*** in the acute phase of cerebral ischemia employing the gerbil model of common carotid artery occlusion.

### MATERIALS AND METHODS

#### Animals and Preparation of Ischemic Insults

One hundred and forty male Mongolian gerbils (12–15 weeks; 70–90 g) were provided by the National Center for Laboratory Animal Breeding (CENPALAB, Havana, Cuba) and adapted to experimental conditions for 7 days. The animals were maintained in controlled environmental rooms at 22 ± 2°C, relative humidity 55–60%; light-dark cycle was 12 h/12 h and there were 15–20 room air changes per hour. All materials used to maintain the animals were autoclaved at 121°C for 20 min. Food and water was provided *ad libitum*. Each protocol was discussed and approved by the Institutional Ethics Committee, considering the international standards established by ICLAS[26]. The animals were distributed according to a completely randomized design in four groups of 35 animals each:

- Group I: Control group.
- Group II: Unilateral permanent ischemia with nasal vehicle
- Group III: Unilateral permanent ischemia coupled with ***IN*** Neuro-EPO treatment (Neuro-EPO ***IN***)
- Group IV: Unilateral permanent ischemia coupled with ***IP*** treatment of rHu-EPO (EPO ***IP***)

rHu-EPO (**ior®CIM** from CIMAB SA, Havana, Cuba) and Neuro-EPO (not a commercial product; the patent is PCT/cu2006/000001 Patent 20050138) were supplied by the Center of Molecular Immunology (CIM, Havana, Cuba) and diluted in PBS (pH 7.0) at 0.15 mM.

***IN*** administration was performed essentially as described previously[27], taking into account the established *Guide to the Care and Use of Experimental Animals*[26]. Immediately after surgery, the gerbils were placed on their backs and a total of 10 μg (249.4 UI) of Neuro-EPO or a corresponding volume of vehicle solution per gerbil was given in nose drops (5 μl/drop) over a 1- to 2-min period, alternating drops between the left and right nares. The mouth and the opposite naris were closed during the administration so the drops could be naturally inhaled high into the nasal cavity. Neuro-EPO ***IN***, a corresponding volume of vehicle ***IN***, and EPO ***IP*** total dose 5000 UI EPO were administered three times per day during 4 days, beginning immediately after surgery.

### Surgical Procedure and Spontaneous Exploratory Activity Measurement

Gerbils were anesthetized with ketamine-atropine-diazepam (47, 0.02, and 5 mg/kg, respectively). Lesions were performed according to Butterfield and McGraw's method[28]. Briefly, the right common carotid artery (CCA) was isolated, double ligated using silk 5-0 suture, and sectioned. In control operated animals, the artery was only isolated.

Twenty-four hours after unilateral permanent ischemia, the appearance of the following clinical signs of infarction was assessed: palpebral ptosis, bristling, loss of tone and reflexes in the four limbs, postural asymmetry, rolling or circling, and death. Each sign was scored separately. The sum of scores creates a general neurological score for each animal[29]. The clinical signs of brain infarction were assessed as previously established[27]. In brief, each animal was evaluated to determine its neurological state according to a scale (with a maximum of 30 and a minimum of 0), grasping strength, and disturbances in posture and gait.

Gerbils were placed in the center of a round open field (30 cm diameter and 25 cm high). "Rearing" was considered as standing straight up on the hind limbs and tail, until the animal returned any forelimb to the floor or touched the open field wall with any forelimb. Neither a subsequent straight after a rearing without reaching the floor with any forepaw nor kangaroo-like posture was considered. Exploratory activity was determined by the rearing counted at 3, 6, and 9 min in the open field. An average of the total rearing counts was calculated for each group, rendering three dots per trial. Dots were plotted, and the line obtained by the minimal square method was calculated and considered as the habituation curve. The slope of the habituation curve was used to characterize the animal's state.

### Tissue Preparation

- Histology: gerbils were anesthetized for cardiac perfusion. Each animal was perfused with 20 ml of saline solution and fixed with 60 ml of 4% buffered formaldehyde solution, pH 7.0. Brains were carefully removed and postfixed in the same solution for several days. Fixed brains were dehydrated and embedded in paraffin. Serial coronal sections of 8-μm width were obtained. Sections were stained with hematoxilin-eosin and described using light microscopy.
- Semi-quantitative analysis: a semi-quantitative scale was constructed to evaluate the histological state of each region. The following regions were considered in each hemisphere: hippocampus (CA1, CA2, CA3, CA4 regions), thalamus, caudate-putamen, parietal and temporal cortex. According to the histological aspect, each region was assigned between 0 and 3 points, equivalent to no damage (0), slight (1), moderate (2), and severe (3). The score attained by each animal was denominated grade histology (GH). An animal could reach a maximum GH of 21 and a minimum of 0 for each hemisphere.
- Quantitative morphological analysis: preparations containing dorsal hippocampus (from 1.3 to 1.6 after Bregma), according to the atlas by Loskota et al.[30], were used. Observers were blind to the preparation origin. Pictures of 400× magnifying power were digitized and processed by the ImageJ program (http://rsb.info.nih.gov/ij/, NIH). The area of hippocampus CA1 pyramidal layer was measured, subtracting the area occupied by pyknotic cells. Then, the normal remaining area of CA 1 subfields was determined.

### Statistical Analysis

The log rank test using GraphPad Prism 4 to evaluate survival percentage for the three treated groups and control was employed. Frequencies of clinical signs and rearing counts were analyzed employing the Chi-square independence test. The Mann-Whitney U test was used for comparisons between the control and others groups of neurological score rearing counts, slope, and remaining CA1 area. The Wilcoxon matched pair test was used for comparisons between hemispheres. For correlations between morphological and functional variables, the Spearman's rank correlation coefficient was used. The statistical analysis was carried out according to the Microsoft STATISTICA version 6.0 program. In all, a level of significance of $p < 0.05$ was accepted.

## RESULTS

### Survival

Survival percentage for the three treated groups was significantly lower than for the control group, but the survival of the group treated with Neuro-EPO ***IN*** was higher than that of animals treated with vehicle and EPO ***IP,*** although comparison with the latter did not achieve statistical significance (Fig. 1).

### Clinical Signs of Stroke

The neurological score in the treated groups (Neuro-EPO ***IN*** and EPO ***IP***) was significantly smaller than in the vehicle group, but there was no difference between the two treated groups (Fig. 2)

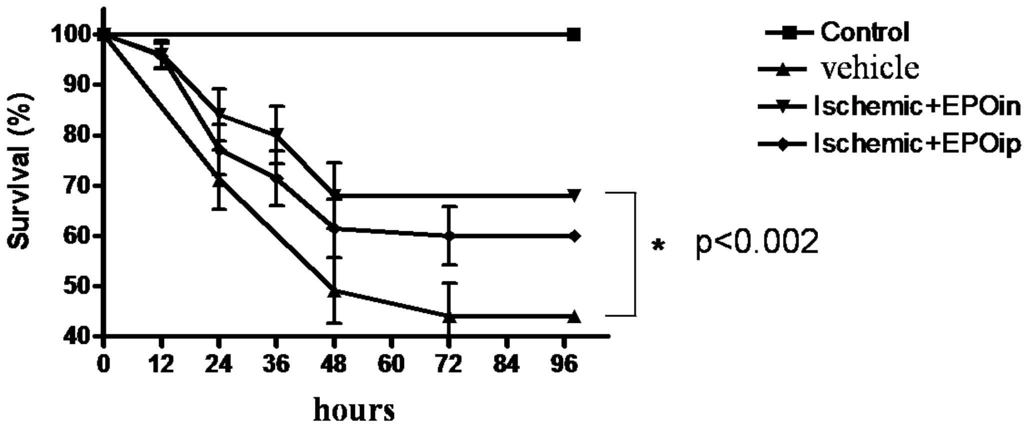

**FIGURE 1**. Survival at 7 days from the permanent occlusion of the common right carotid of the gerbils (n = 35 per group). Vehicle was significantly different from Neuro-EPO (*$p$ < 0.002) in the log rank test.

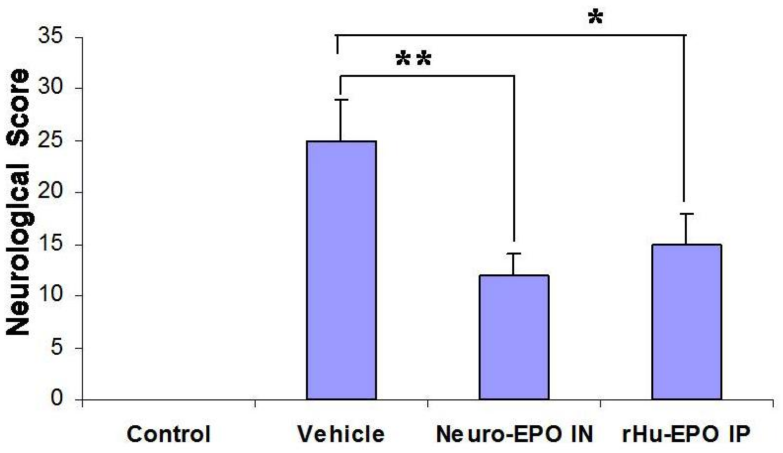

**FIGURE 2**. Neurological score in the pattern of permanent occlusion of the right carotid in Mongolian gerbils. Asterisk indicates a significant difference (*$p$ <0.05; **$p$ < 0.01) (n = 35 for group).

### Spontaneous Exploratory Activity

Analyzing the slopes of the habituation curves in the open field test (Fig. 3), it could be established that in all the experimental groups, except the group tested with the vehicle, significant differences existed between the habituation curves before and after carotid occlusion. These differences in the control group and in the group treated with Neuro-EPO ***IN*** were positive (greater slope after the operation), indicating that these animals were able to recognize the field, while in the groups treated with the vehicle and EPO ***IP***, it was negative (smaller slope in the habituation curve after the operation), indicating a lack of recognition of the previously explored field.

### Qualitative Study

The control group showed no histological lesions of cerebral ischemia in the regions studied. In the ischemic group treated with the vehicle, a large stroke area was observed in the hemisphere ipsolateral to

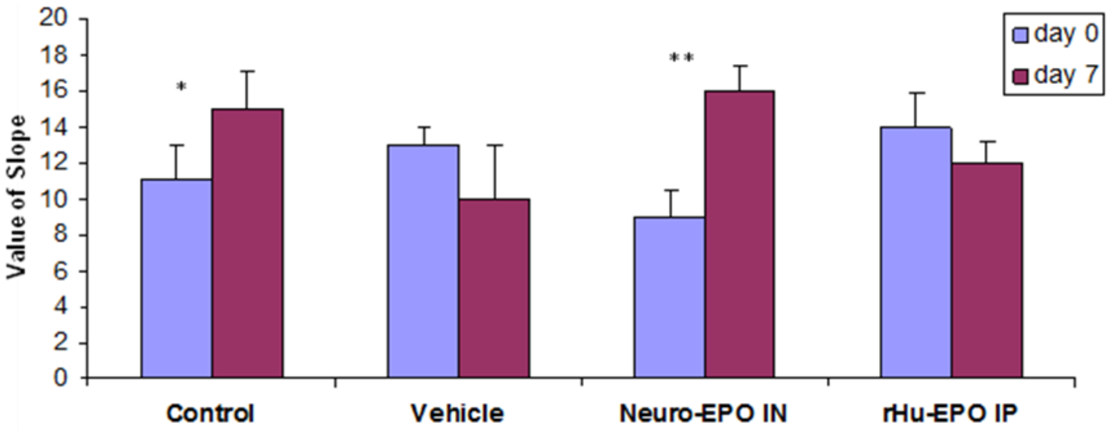

**FIGURE 3.** Slope values before and 7 days after the permanent occlusion of the right common carotid in the Mongolian gerbil. Asterisk indicates a significant difference between 0 and 7 days after lesion (*$p < 0.05$; **$p < 0.01$) (n = 35 for group).

the occlusion of the carotid at 7 days after injury. The stroke area was identified by the death of most of the cellular elements, and presence of cellular remains and of partially empty areas. The stroke area extended to all the regions of the hippocampus, to the parietal and temporal cortex, to nuclei of the thalamus and caudate-putamen. In sectors CA1 and CA3 of the hippocampus, pyramidal pyknotic neurons were observed with acidophilius cytoplasm. Many cells were observed in cariorrexis processes, cariolisis and others in a state of “ghost cells.” In the stroke area, full granulate-greasy corpuscles with products of degradation of myelin were observed. In the caudate-putamen nuclei of both hemispheres, extensive areas of stroke were observed with the presence of abundant heaps of cells, polyploidy, processes in mitosis, also absence of the neuropil and of all the cellular elements.

In the ischemic group treated with EPO ***IP***, irreversible damage in the CA1 and CA2 regions of the hippocampus was detected, and these lesions were characterized by selective neuronal death and increase in the number of microglía. The pyramidal neurons of the hippocampus were eosinophilic and the existence of empty spaces revealed possible neuronal loss. Protection of the CA3 region of the hippocampus was observed. Stroke areas were observed in the parietal cortex and caudate-putamen nucleus, with protection of the histological structure of the temporal cortex. Cell polyploidy and processes in mitosis were observed in the thalamus (Figs. 4 and 5).

In the ischemic group treated with Neuro-EPO ***IN,*** there was slight damage in the CA1 and CA2 regions of the hippocampus, while in the CA3 region, the pyramidal neurons remained normal; isolated areas of stroke were observed in the parietal cortex, with slight pyknosis. In the temporal cortex and thalamus, conservation of the histological structures prevailed in all the layers. The stroke area was less extensive in the ischemic group treated with Neuro-EPO ***IN*** than in the ischemic animals treated with EPO ***IP*** and with the vehicle (Figs. 4 and 5).

### Semiquantitative Analysis

Semiquantitative evaluation of the histological scale showed that animals treated with Neuro-EPO ***IN*** displayed a lower histological grade than the ischemic animals treated with the vehicle, fundamentally at the expense of CA3, thalamus, and temporal cortex of the ipsilateral hemisphere. On the other hand, animals treated with EPO ***IP*** had a significantly lower histological grade in the CA3 region and temporal cortex with respect to those treated with the vehicle, not finding significant differences with any other region (Table 1).

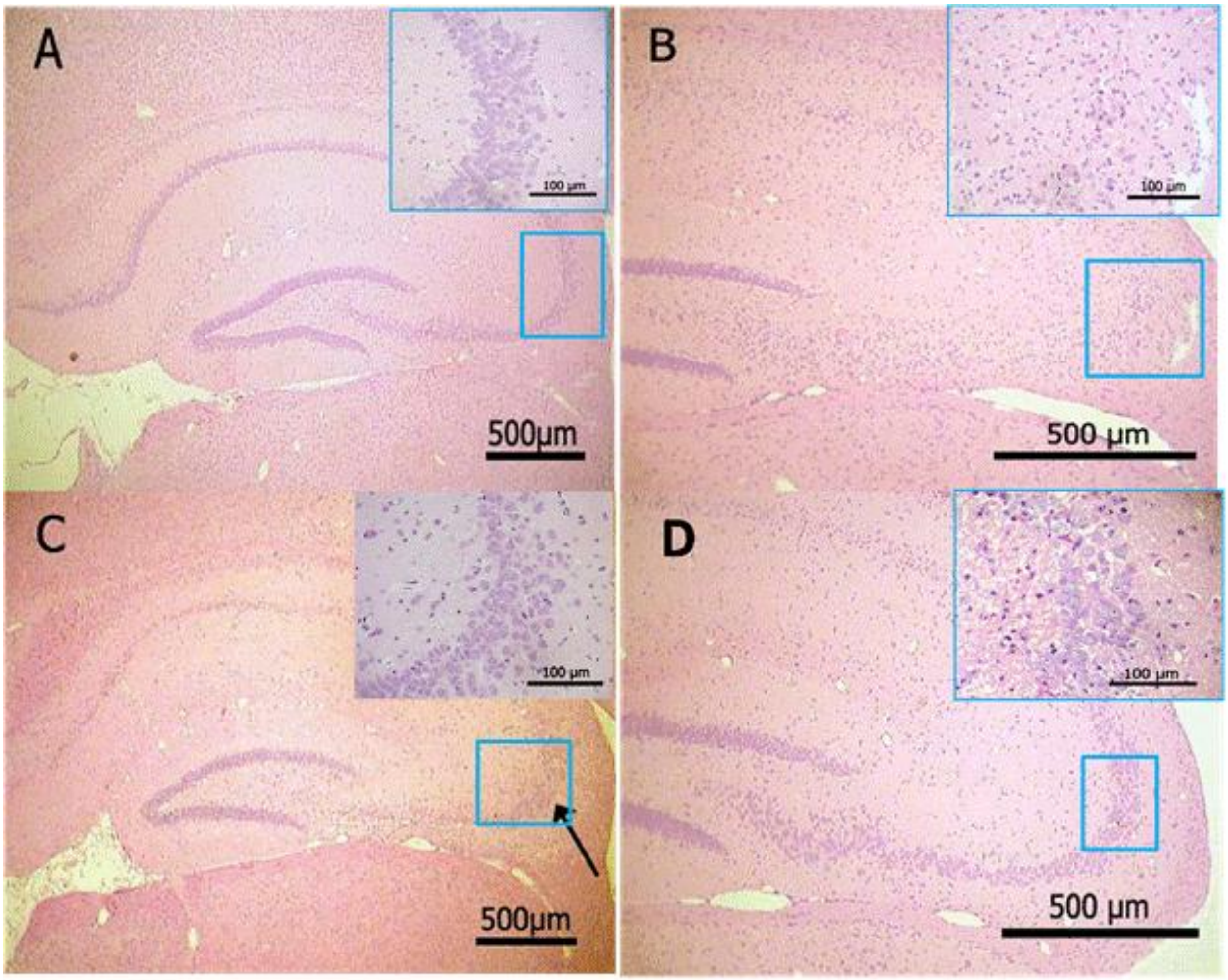

**FIGURE 4**. H-E. (A) Normal dorsal hippocampus. CA3 region amplified. (B) Ischemia stroke in the hippocampus 7 days after occlusion. CA3 region amplified with pyramidal pyknotic neurons. (C) Neuro-EPO ***IN*** protects sector CA3 of the dorsal hippocampus and CA1 is not protected. Arrow: conservation of CA3 neurons. (D) EPO ***IP*** protects sector CA3 of the dorsal hippocampus and CA1 is not protected.

### Quantitative Analysis

The number of healthy neurons in the contralateral hemispheres (left) was the same in the four groups ($p > 0.05$). In the hemisphere ipsilateral to the occlusion, the number of healthy neurons in CA1 was significantly smaller in the groups where the carotid was injured, i.e., in those treated with vehicle, Neuro-EPO ***IN***, and EPO ***IP***, than in the control group (Table 2).

### DISCUSSION

The present study is the first to investigate the effect of Neuro-EPO ***IN*** vs. EPO ***IP*** on behavioral and histological variables in focal ischemia in gerbils. Previous to this work, only a few publications appeared in MEDLINE evaluating rHu-EPO *in vivo* as a neuroprotector in cerebral ischemia models in gerbils[31,32,33,34,35]. The routes employed to deliver EPO to the damaged brain were ***IP***[33], ***ICV***[34,36], and/or ***IP-ICV***[37].

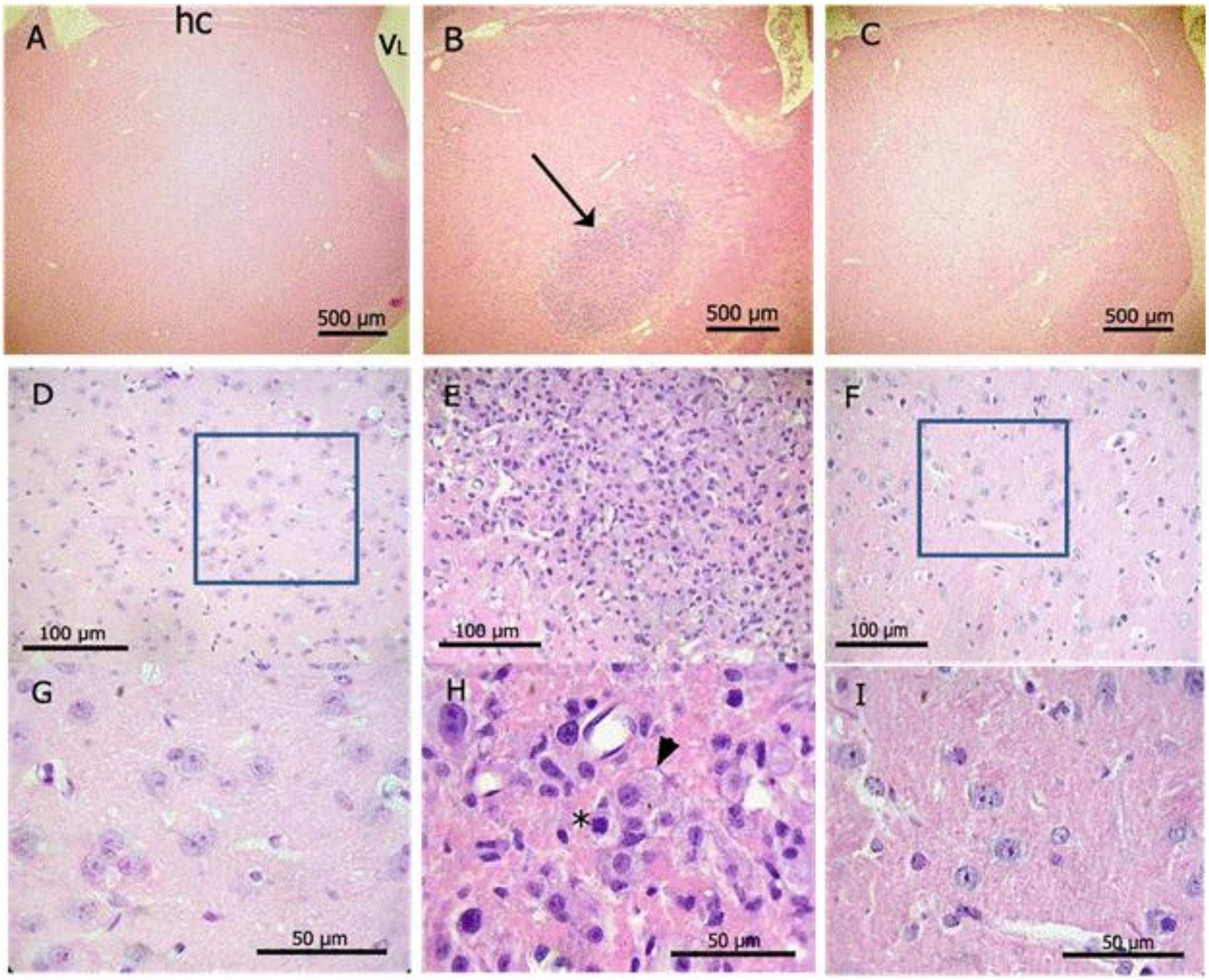

**FIGURE 5**. H-E. (A) Control panoramic view of ipsilateral thalamus. VL: lateral ventricle; hc: hippocampus. (B) Unilateral ischemia producing big quantities of macrophages in nuclei of the thalamus. Arrow: macrophages. (C) Diminished damage in thalamus with Neuro-EPO ***IN***. (D) Control ipsilateral thalamus (lower magnification). (E) Macrophage nuclei are shown. (F) Aspect similar to the control. (G) Control ipsilateral thalamus (higher magnification). (H) Macrophage with cytoplasm vacuolado (arrow head); macrophage in mitosis (asterisk). (I) Aspect similar to the control.

**TABLE 1**
**Semi-Quantitative Evaluation of the Histological Scale**

| Region | | Control | Vehicle *IN* | Neuro-EPO *IN* | rHu-EPO *IP* |
|---|---|---|---|---|---|
| Hippocampus | CA 1 | 0.1 | 3 | 2 | 1.7 |
| | CA 2 | 0.1 | 2.4 | 1 | 0.8 |
| | CA 3 | 0 | 2.8 | 0.3* | 0.7* |
| | CA 4 | 0.1 | 1.8 | 0.7 | 0.6 |
| Thalamus | | 0 | 2.2 | 0.3* | 1.2 |
| Caudate-putamen | | 0 | 2.4 | 0.8 | 1.3 |
| Cortex parietal | | 0 | 2.2 | 0.8 | 1.1 |
| Cortex temporal | | 0 | 2.2 | 0.2* | 0.7* |
| Grade histology | | 0.2 | 19 | 6.2* | 7.3* |

*$p < 0.05$ with regard to vehicle IN. Tests of Kruskal-Wallis and Mann-Whitney.

**TABLE 2**
**Linear Density of Healthy Neurons in Regions of the Hippocampus**

| Hippocampus | Control | | Vehicule *IN* | | Neuro-EPO *IN* | | EPO *IP* | |
|---|---|---|---|---|---|---|---|---|
| | HL | HR | HL | HR | HL | HR | HL | HR |
| CA 1 | 229 ± 21 | 232 ± 17 | 239 ± 34 | 155 ± 107 | 221 ± 23 | 174 ± 85 | 227 ± 22 | 132 ± 98 |

Average values in neurons/mm. HL: left hemisphere; HR: right hemisphere. Kruskal-Wallis and Mann-Whitney. There were no differences between groups.

Nasal delivery of therapeutic proteins to the CNS clearly involves extraneuronal transport as it occurs in minutes rather than hours. It is an attractive possibility to use in acute stroke where "time *is brain*". In a previous paper, we showed that Neuro-EPO ***IN*** reached the CNS in pharmacological concentration in only 5 min[27]. This result in rodents was confirmed with a pharmacokinetic study in nonhuman primates with ***IN*** application of Neuro-EPO. In this same study, the ***IN*** administration of Neuro-EPO resulted in 3.5 times higher concentration of CSF EPO levels of times faster that the administration of ***IV*** rHu-EPO[23]. We previously demonstrated the presence of I125-labeled Neuro-EPO in the brain after an intranasal single-dose application, strongly suggesting the passage of the molecule to the brain regions not anatomically related[27,38]. This supports the findings of other authors and our previous reports on the neuroprotector effect of applied ***IN*** EPO in stroke models[16,21].

Mortality in this model of stroke reflects the intensity of the lesion and it is an important indicator for the evaluation of drugs in the treatment of ischemia. For this model, rates of mortality between 30 and 40% at 24 or 48 h from the lesion, respectively, have been reported[39,40], agreeing with our results. Survival of the group treated with Neuro-EPO ***IN*** in this work was higher than that of animals treated with the vehicle and EPO ***IP,*** confirming our previous reports on the neuroprotective effect of Neuro-EPO ***IN***[24,27,41].

To assess the mechanisms through which nasal Neuro-EPO achieves its neuroprotective effect is beyond the scope of this work, but we speculate that they are probably related toh the same mechanisms described for rHu-EPO or its derivatives CEPO or sialo EPO in different models of stroke: antiapoptotic[42,43,44,45], anti-inflammatory[46,47], neurogenic[48,49], angiogenic[15,50,51,52], and antioxidant[53,54,55,56]. Further animal experiments of Neuro-EPO ***IN*** on focal ischemia models are necessary in order to understand the mechanisms underlying improved survival.

Animals with more severe clinical signs of stroke corresponded with two groups: vehicle and EPO ***IP***. In these cases, the remarkable lack of rearing in a typical active rodent is due to motor, rather than to other behavioral impairments. According to open field performance, these groups had a significantly depressed exploratory activity compared to Neuro-EPO ***IN*** before and after 7 days of ischemia and treatments.

These results suggest the existence of a subtle functional alteration in this subset of animals, probably at the synaptic level, undetectable with hematoxylin-eosin staining.

The histological analysis demonstrated that Neuro-EPO ***IN*** produced neuroprotection in the CA3 region of the hippocampus, thalamus, and temporal cortex, regions that constitute the frontiers of stroke, and mainly in the region of the thalamus, while the application of EPO ***IP*** did not. Neuro-EPO ***IN*** could be related with the improved learning found in the open field test.

According to the open field test, we conclude that the animals treated with vehicle and with EPO ***IP*** present cognitive dysfunction associated with ischemia and especially to the CA1 sector of the hippocampus that was seriously damaged, the most vulnerable area to ischemic damage, which regulates and controls learning and memory. These animals do not remember the environment explored 7 days after having explored it for the first time, thus they continue zooming with the same frequency in the three intervals of time. However, the animals treated with Neuro-EPO ***IN*** present a smaller damage, which does not affect their learning or memory.

Other authors demonstrated that ***ICV*** administration of EPO increased the natatorium capacity, and diminished the area of cortical stroke and thalamic degeneration in rats with permanent focal ischemia[37]. In our work, the structures where neuroprotection was observed were CA3, thalamus, and temporal cortex localized at the periphery of the stroke area, and probably constituted the area of ischemic penumbra during the first hours after the occlusion. Previous results with Neuro-EPO ***IN*** in medial cerebral artery occlusion show a significant reduction in the infarcted area in animals subjected to focal ischemia compared with animals treated with the vehicle of the formulation and rHu-EPO ***IP***[38].

It is considered that in the ischemic penumbra area, a blood flow below normal levels still exists[57]. Probably, the arrival of Neuro-EPO to the region of ischemic penumbra protected those cells against phenomena like excitotoxicity and apoptosis[14]. Also, it has been demonstrated that EPO stimulates angiogenic and neurogenic processes[11,15,58], providing a favorable microenvironment for neuronal plasticity during the recovery from stroke. In this work, during the 7 days of ischemia, histological damage was diminished in the above-mentioned areas.

In the CA1 region of the hippocampus, good neuroprotection was not observed, a finding corroborated with neuron counting. This area of the hippocampus is the most sensitive to ischemia[59], for which the neuroprotective effect of any drug would have lower possibilities of being evidenced than in less vulnerable areas. Animals from the ischemic group treated with the vehicle presented necrosis of the parietal and temporal cortex, hippocampus, thalamus, and caudate-putamen; loss of pyramidal neurons of CA1; and cognitive deterioration; findings similar to those described in other works[60,61]. These negative effects were reduced by the pharmacological effect of Neuro-EPO ***IN***.

The results of this work constitute an indirect evidence of the passage of Neuro-EPO to the CNS through the nasal cavity. The nasal route gives the possibility of administering much lower doses than those necessary by the ***IV*** route[16]. These results suggest that Neuro-EPO ***IN*** can have a therapeutic effect on stroke damage after focal cerebral ischemia, applying a dose that is lower than those previously reported for any recombinant EPO.

Taken together, all these results suggest that Neuro-EPO ***IN*** is a better neuroprotectant than EPO ***IP***, at least in this model of stroke.

The therapeutic strategy of developing the nasal delivery of Neuro-EPO could allow for its multiple and chronic use in neurodegenerative disorders.

In conclusion, ours results using Neuro-EPO ***IN*** suggest that it could be a potential novel neurotherapeutic approach for the treatment of ischemic stroke. The above-mentioned studies also indicate the safety and proof-of-concept of the initiation of clinical trials in stroke patients.

### DISCLOSURE

Drs. García Rodríguez and Sosa Testé are coinventors on the patent “rh-epo nasal formulations with low sialic acid concentration for the treatment of diseases of the central nervous system”, cu. Patent Formulation International Application No.: PCT/CU2006/000007 OCPI, Havana, Cuba. The authors report no other conflict of interest concerning in this paper.

### ACKNOWLEDGMENTS

We thank Charles M. Hollingshead, University of Guelph, for critical reading of this manuscript.

## References

1. MINSAP (2003) Anuario Estadistico de Salud en Cuba. Available from www.infomed.sld.cu/servicios/estadisticas/
2. Ovbiagele, B., Kidwell, C.S., Starkman, S., and Saber, J.L. (2003) Neuroprotective agents for the treatment of acute ischemic stroke. *Curr. Neurol. Neurosci. Rep*. **3,** 9–20.
3. Lipton, P. (1999) Ischemic cell death in brain neurons. *Physiol. Rev.* **79(4),** 1431–568.
4. Siren, A.L., Fasshauer, T., Bartels, C., and Ehrenreich, H. (2009) Therapeutic potential of erythropoietin and its structural or functional variants in the nervous system. *Neurotherapeutics* **6(1),** 108–127.
5. DeBow, S.B., Clark, D.L., Maclellan, C.L., and Colbourne, F. (2003) Incomplete assessment of experimental cytoprotectants in rodent ischemia studies. *Can. J. Neurol. Sci*. **30(4),** 368–374.
6. García Salman, J.D. (2004) Protección neuronal endógena: un enfoque alternativo. *Rev. Neurol.* **38(2),** 150–155.
7. Dirnagl, U., Simon, R.P., and Hallenbeck, J.M. (2003) Ischemic tolerance and endogenous neuroprotection. *Trends Neurosci.* **26(5)**, 248–254.
8. Jelkmann, W. (2004) Molecular biology of erythropoietin. *Intern. Med*. **43,** 649–659.
9. Kalialis, L.V. and Olsen, N.V. (2003) Erythropoietin--a new therapy in cerebral ischemia? *Ugeskr. Laeger* **165**, 2477.
10. Eid, T. and Brines, M. (2002) Recombinant human erythropoietin for neuroprotection: what is the evidence? *Clin. Breast Cancer* **Suppl 3,** 109–115.
11. Marti, H.H., Bernaudin, M., Petit, E., and Bauer, C. (2000) Neuroprotection and angiogenesis: dual role of erythropoietin in brain ischemia. *News Physiol. Sci.* **15(5),** 225–229.
12. Lipšic, E., Schoemaker, R.G., van der Meer, P., Voors, A.A., van Veldhuisen, D., and van Gilst, W.H. (2006) Protective effects of erythropoietin in cardiac ischemia: from bench to bedside. J. Am. Coll. Cardiol. **48(11),** 2161–2167.
13. Bernaudin, M., Marti, H.H., Roussel, S., et al. (1999) A potential role for erythropoietin in focal permanent cerebral ischemia in mice. *J. Cereb. Blood Flow Metab.* **19(6),** 643–651.
14. Siren, A.L., Fratelli, M., Brines, M., et al. (2001) Erythropoietin prevents neuronal apoptosis after cerebral ischemia and metabolic stress. *Proc. Natl. Acad. Sci. U. S. A*. **98(7),** 4044–4049.
15. Wang, L., Zhang, Z., Wang, Y., Zhang, R., and Chopp, M. (2004) Treatment of stroke with erythropoietin enhances neurogenesis and angiogenesis and improves neurological function in rats. *Stroke* **35(7),** 1732–1737.
16. Garcia Rodriguez, J.C. and Sosa Teste, I. (2009) The nasal route as a potential pathway for delivery of erythropoietin in the treatment of acute ischemic stroke in humans. *TheScientificWorldJOURNAL* **9,** 970–981.
17. Yamashita, T., Nonoguchi, N., Ikemoto, T., Miyatake, S., and Kuroiwa, T. (2010) Asialoerythropoietin attenuates neuronal cell death in the hippocampal CA1 region after transient forebrain ischemia in a gerbil model. *Neurol. Res.* **32(9),** 957–962.
18. Hermann, D.M. (2009) Enhancing the delivery of erythropoietin and its variants into the ischemic brain. *TheScientificWorldJOURNAL* **9,** 967–969.
19. Hanson, L.R. and Frey, W.H. (2008) Intranasal delivery bypasses the blood-brain barrier to target therapeutic agents to the central nervous system and treat neurodegenerative disease. *BMC Neurosci*. **9(Suppl 3),** S5.
20. Pathan, S.A., Iqbal, Z., Zaidi, S.M., et al. (2009) CNS drug delivery systems: novel approaches. *Recent Pat. Drug Deliv. Formul.* **3(1),** 71–89.
21. Fletcher, L., Kohli, S., Sprague, S.M., et al. (2009) Intranasal delivery of erythropoietin plus insulin-like growth factor-I for acute neuroprotection in stroke. *J. Neurosurg.* **111(1),** 164–170.
22. Thorne, R.G., Pronk, G.J., Padmanabhan, V., and Frey, W.H. (2004) Delivery of insulin-like growth factor-I to the rat brain and spinal cord along olfactory and trigeminal pathways following intranasal administration. *Neuroscience* **127(2),** 481–496.
23. Sosa, I., Cruz, J., Santana, J., et al. (2007) Paso de la molécula de eritropoyetina humana recombinante con bajo contenido de ácido siálico al Sistema nervioso central por la vía intranasal en los modelos del *meriones unguiculatus* y el primate no humano *Macaca fascicularis. Rev. Salud Anim*. **29(2),** 1–6.
24. Subiros Martínez, N., García Rodríguez, J.C., González Navarro, B., Sosa, I., and García Salman, J.D. (2005) Evaluación histológica del efecto de la Eritropoyetina vía intranasal, sobre la muerte neuronal retardada en gerbos sometidos a isquemia cerebral transitoria. Estudio preliminar. 7º Congreso Virtual Hispanoamericano de Anatomía Patológica 2005. Available from www.conganat.org/7congreso/445.pdf
25. Yu, Y.-P., Xu, Q.-Q., Zhang, Q., Zhang, W.-P., Zhang, L.-H., and Wei, E.-Q. (2005) Intranasal recombinant human erythropoietin protects rats against focal cerebral ischemia. *Neurosci. Lett.* **387,** 5–10.
26. CCAC (1993) *Guide to the Care and Use of Experimental Animals*. 2nd ed. Canadian Council on Animal Care, Ontario, Canada. 211 p.
27. Sosa, I., García Rodríguez, J.C., Santana, J., et al. (2006) Intranasal administration of recombinant human erythropoietin exerts neuroprotective effects on post-ischemic brain injury in Mongolian gerbils. *PharmacologyOnline* **1,** 100–112.
28. Butterfield, J.D. and McGraw, C.P. (1978) Effect of the DPPD (diphenyl-para-phenylendiamine) on stroke and cerebral edema in gerbils. *Stroke* **9(5),** 480–483.

29. Lawner, P., Laurent, J., Simeone, F., Fink, E., and Rubin, E. (1979) Attenuation of ischemic brain edema by pentobarbital after carotid ligation in the gerbil. *Stroke* **10(6),** 644–647.
30. Loskota, W.J., Lomax, P., and Verity, M.A. (1973) *A Stereotaxic Atlas of the Mongolian Gerbil Brain (Meriones unguiculatus)*. Ann Arbor Science, Los Angeles.
31. Illum, L. (2002) Nasal drug delivery: new developments and strategies. *Drug Discov. Today* **7(23),** 1184–1189.
32. Pardridge, W.M. (2007) Blood-brain barrier delivery. *Drug Discov. Today* **12(1–2),** 54–61.
33. Faure, S., Oudart, N., Javellaud, J., Fournier, A., Warnock, D., and Achard, J. (2006) Synergistic protective effects of erythropoietin and olmesartan on ischemic stroke survival and post-stroke memory dysfunctions in the gerbil. *J. Hypertens*. **24(11),** 2255–2261.
34. Wen, T.C., Sadamoto, Y., Tanaka, J., et al. (2002) Erythropoietin protects neurons against chemical hypoxia and cerebral ischemic injury by up-regulating Bcl-xL expression. *J. Neurosci. Res.* **67(6),** 795–803.
35. Lapchak, P.A. (2010) Erythropoietin molecules to treat acute ischemic stroke: a translational dilemma! *Expert Opin. Investig. Drugs* **19(10),** 1179–1186.
36. Catania, M.A., Marciano, M.C., Parisi, A., et al. (2002) Erythropoietin prevents cognition impairment induced by transient brain ischemia in gerbils. *Eur. J. Pharmacol*. **437(3),** 147–150.
37. Sadamoto, Y., Igase, K., Sakanaka, M., et al. (1998) Erythropoietin prevents place navigation disability and cortical infarction in rats with permanent occlusion of the middle cerebral artery. *Biochem. Biophys. Res. Commun.* **253,** 26–32.
38. Nuñez, Y., Bueno, P.V., Carrillo, D.C., et al. (2009) Neuroprotective effect of a nasal formulation of erythropoietin with low sialic acid content. *Rev. Cubana Farm.* **43(1),** 1–13.
39. Kirino, T. (1982) Delayed neuronal death in the gerbil hippocampus following ischemia. *Brain Res.* **239(1),** 57–69.
40. Sosa, I., García Salman, J.D., Rodríguez, Y., Subiros, N., Rodríguez, K., and Thomas, A. (2007) Sensibilidad de los modelos de isquemia global y focal en el gerbil de Mongolia. Available from www.Ilustrados.com:EyFpAVAlpCfBOoWnY
41. Sosa, I., Mengana, T.Y., García, S.J.D., et al. (2008) Recombinant human erythropoietin as a neuroprotective therapy in brain ischemia. *Biotecnol. Apl.* **25,** 223–229.
42. Brines, M.L. and Cerami, A. (2008) Erythropoietin-mediated tissue protection: reducing collateral damage from the primary injury response. *J. Intern. Med.* **264(5),** 405–432.
43. Ghezzi, P. and Brines, M. (2004) Erythropoietin as an antiapoptotic, tissue-protective cytokine. *Cell Death Differ*.**11(Suppl 1),** S37–44.
44. Mattson, M.P., Culmsee, C., and Yu, Z.F. (2000) Apoptotic and antiapoptotic mechanisms in stroke. *Cell Tissue Res.* **301,** 173–187.
45. Yang, X.F., Xiong, J.W., Wang, Z.L., et al. (2009) [Mechanism of inhibition of apoptosis of human umbilical vein endothelial cells induced by oxidized-low density lipoprotein by erythropoietin]. *Zhongguo Wei Zhong Bing Ji Jiu Yi Xue* **21(11),** 656–659.
46. Agnello, D., Bigini, P., Villa, P., et al. (2002) Erythropoietin exerts anti-inflammatory effect on the CNS in a model of experimental autoinmune encephalomyelitis. *Brain Res*. **952,** 128–134.
47. Whitehead, S.N., Hachinski, V.C., and Cechetto, D.F. (2005) Interaction between a rat model of cerebral ischemia and beta-amyloid toxicity: inflammatory responses. *Stroke* **36,** 107–112.
48. Arcasoy, M.O. (2008) The non-haematopoietic biological effects of erythropoietin. *Br. J. Haematol.* **141(1),** 14–31.
49. Brines, M.L. and Cerami, A. (2006) Discovering erythropoietin's extra-hematopoietic functions: biology and clinical promise. *Kidney Int*. **70(2),** 246–250.
50. Buemi, M., Galeano, M., Sturiale, A., et al. (2004) Recombinant human erythropoietin stimulates angiogenesis and healing of ischemic skin wounds. *Shock* **22(2),** 169–173.
51. Li Y, Lu Z, Keogh CL, Yu SP, and Wei L. (2007) Erythropoietin-induced neurovascular protection, angiogenesis, and cerebral blood flow restoration after focal ischemia in mice. J Cereb Blood Flow Metab May;**27(5),** 1043–54.
52. Liu, S.P., Lee, S.D., Lee, H.T., et al. (2010) Granulocyte colony-stimulating factor activating HIF-1alpha acts synergistically with erythropoietin to promote tissue plasticity. *PLoS One* **5(4),** e10093.
53. Castillo, J., Varez-Sabin, J., Davalos, A., et al. (2003) [Consensus review. Pharmacological neuroprotection in cerebral ischemia: is it still a therapeutic option?]. *Neurologia* Sep;**18(7),** 368–384.
54. Chan, P.H. (2001) Reactive oxygen radicals in signaling and damage in the ischemic brain. *J. Cereb. Blood Flow Metab*.**21(1),** 2–14.
55. Ikeda, K., Negishi, H., and Yamori, Y. (2003) Antioxidant nutrients and hypoxia/ischemia brain injury in rodents. *Toxicology* **189(1–2),** 55–61.
56. Margaill, I., Plotkine, M., and Lerouet, D. (2005) Antioxidant strategies in the treatment of stroke. *Free Radic. Biol. Med*. **39(4),** 429–443.
57. Phan, T.G., Wright, P.M., Markus, R., Howells, D.W., Davis, S.M., and Donnan, G.A. (2002) Salvage the ischaemic penumbra: more than just reperfusion? *Clin. Exp. Pharmacol.* **29,** 1–10.
58. Shingo, T., Sorokan, S.T., Shimazaki, T., and Weiss, S. (2001) Erythropoietin regulates the in vitro and in vivo production of neuronal progenitors by mammalian forebrain neural stem cells. *J. Neurosci.* **21(24),** 9733–9743.
59. Kirino, T. and Sano, K. (1984) Selective vulnerability in the gerbil hippocampus following transient ischemia. *Acta Neuropathol. (Berl.)* **62(3),** 201–208.

60. Payan, H.M. and Conrad, J.R. (1977) Carotid ligation in gerbils. Influence of age, sex, and gonads. *Stroke* **8(2),** 194–196.

61. Torup, L. and Leist, M. (2006) Development of non-erythropoietic erythropoietin variants for neuroprotection. In *Erythropoietin and the Nervous System*. Höke, A., Ed. Springer, New York.
